# Chronic Neurobehavioral and Neuropathological Consequences of Repeated Blast Exposure in P301S Transgenic Tau Rats

**DOI:** 10.1089/neur.2024.0168

**Published:** 2025-04-29

**Authors:** Claire Robey, Antigone Grillakis, Anya Fan, Jiong Liu, Laura B. Tucker, Amanda H. Fu, Yeonho Kim, Joseph T. McCabe

**Affiliations:** ^1^ Department of Anatomy, Physiology, and Genetics, Uniformed Services University of the Health Sciences, Bethesda, Maryland, USA.; ^2^ Henry M. Jackson Foundation for the Advancement of Military Medicine, Inc., Bethesda, Maryland, USA.; ^3^ Department of Laboratory Animal Resources, Preclinical Behavior and Modeling Core, Uniformed Services University of the Health Sciences, Bethesda, Maryland, USA.

**Keywords:** aging, behavior, neuropathology, sex differences, tau, traumatic brain injury

## Abstract

Repeated blast traumatic brain injury (rbTBI) is linked to dementia risk, potentially due to abnormal tau accumulation, although a definitive causal relationship remains elusive. This study aims to develop a model of rbTBI-induced tauopathy. We utilized wild-type (WT) rats and rats that are heterozygous for the mutated P301S human tau gene (Tg12099 +/−), the presence of which increases the propensity to develop tau neuropathology. At 2–3 months of age, rats were exposed to five blasts using the Advanced Blast Simulator or sham procedures. Behavioral and histological outcomes were evaluated at 10 and 15 months post-injury, respectively. The open field test revealed increased activity in blast-injured animals compared with sham. Tg12099 +/− females exhibited greater travel distances than WT females, while male activity levels did not differ by genotype. The novel object recognition test indicated impaired recognition memory in blast-injured animals, which was unrelated to genotype. There was a greater accumulation of phosphorylated tau in several brain regions of Tg12099 +/− rats compared with WT rats, yet no observable blast injury effect. Blast did not alter astro- and microgliosis, but increased astrogliosis was observed in Tg12099 +/− rats compared with WT rats in a region-dependent manner. We observed sex-dependent changes in microgliosis within the Tg12099 +/− group, with male Tg12099 +/− rats exhibiting increased IBA1 immunostaining compared with females. No such sex difference was observed in WT rats. Our findings suggest that while rbTBI can induce persistent behavioral deficits in rats, it does not exacerbate neuropathology in Tg12099 rats.

## Introduction

Traumatic brain injury (TBI) has emerged as a signature injury sustained by U.S. military service members in recent conflicts.^1^ While the majority of these cases are classified as mild and symptoms typically resolve quickly, there is mounting evidence to suggest that repeated exposures to mild TBI can lead to long-term poor outcomes.^2–6^ Furthermore, the widespread use of improvised explosive devices (IEDs) has significantly increased the prevalence of blast-related TBI among soldiers.^7^ Although often considered sub-concussive, there is also a growing concern for the effects of low-level blasts. Occupations that involve low-level, frequent exposures, such as breaching or employing shoulder-fired weapons, have been linked to symptoms of mild TBI, including headache, tinnitus, hearing loss, memory problems, cognitive dysfunction, and mood changes.^8^ The chronicity of these symptoms suggests an underlying degenerative process in the brain of affected individuals.

The relationship between neurodegeneration and TBI has long been studied, where a history of TBI increases the risk of Alzheimer’s disease and other dementias.^9–14^ It has also been demonstrated that this risk occurs through a dose-response relationship, with a greater number of TBIs correlating to a higher likelihood of dementia.^15^ A hallmark of these diseases is the abnormal accumulation and phosphorylation of tau, an essential microtubule-binding protein.^16^ Under normal physiological conditions, tau acts to stabilize the cytoskeleton and plays a key role in axoplasmic transport.^17^ While post-translational modifications, such as phosphorylation, are necessary for the normal function of tau, abnormal phosphorylation can contribute to aggregation and the development of toxic neurofibrillary tangles seen in several neurodegenerative diseases, collectively labeled tauopathies.^18^ Tauopathies have been linked to repeated impact TBI, particularly in professional athletes.^19^ However, whether repeated blast exposure is a risk factor for tauopathy is less clear. One study of career breachers found, in comparison to matched military or civilian law enforcement controls, elevated levels of neuronal-derived extracellular vesicle tau that were associated with neurobehavioral symptoms.^20^ Another study utilized positron emission tomography imaging with a tau ligand [^18^F]AV1451 (flortaucipir) and found excessive retention of the ligand in frontal, parietal, and temporal brain regions in blast-exposed veterans.^21^ However, it is important to consider that blast exposure often occurs alongside impact TBI, as individuals may be thrown into objects or struck by flying debris during a blast.^22^ It is, therefore, challenging to isolate the effects of blast exposure and its relationship to tauopathy.

Animal models offer the unique advantage of isolating the effects of primary blast exposure and conducting longitudinal studies in an accelerated timeframe. Rats exposed to repeated blast TBI (rbTBI) develop cognitive, motor, and anxiety-like behavioral deficits that persist up to 1 year post-exposure.^23–27^ In addition to behavioral changes, neuropathological outcomes have also been identified at acute and chronic timepoints in pre-clinical rbTBI studies, including evidence of micro- and astrogliosis, blood–brain barrier disruptions, neuroinflammation, axonal damage, and neurodegeneration.^21,28,29^ For a comprehensive review on behavior and pathological outcomes in pre-clinical rbTBI studies, refer to Ravula et al.^30^

To better understand the relationship between these chronic changes and tauopathy, several studies have utilized transgenic tau animals in conjunction with a TBI model. Yet, studies to date have been performed using transgenic mice, thus limiting the generalizability of findings.^31–44^ To diversify and extend upon both the TBI and tauopathy model, the present study employed a recently developed transgenic tau rat (Tg12099).^45^ Compared with the mouse model, the rat may be advantageous as rats possess a more complex central nervous system, greater cognitive abilities, and a richer behavioral repertoire.^46,47^ To drive transcription, the human 0N4R isoform with a MAPT*P301S mutation was linked with the rat prion protein promotor.^48^ Both homozygous (Tg12099 +/+) and heterozygous (Tg12099 +/−) rats ubiquitously express human tau (htau) in the CNS.^45^ Tg12099 +/+ rats have been reported to spontaneously develop abnormal accumulations of phosphorylated tau as early as 6 months, which progressively worsen over time, leading to overt neurodegeneration with age.^45^ In addition, Tg12099 +/+ rats have a reported median survival of 14 months, and exhibit signs of neurological decline including ataxia, bradykinesia, and seizures.^45^ In contrast, Tg12099 +/− rats reportedly display very low levels of phosphorylated tau, minimal neuropathologic changes by 18 months of age, and survival extending beyond 24 months.^45^ Importantly, tauopathy can be induced in Tg12099 +/− rats when exogenously administered recombinant tau fibrils or brain homogenates from aged Tg12099 +/+ rats, suggesting these rats are predisposed to develop tauopathy if given the appropriate catalyst.^45^ In the present study, we sought to determine whether rbTBI would trigger the heightened development of tauopathy in Tg12099 +/− rats. Rats were exposed to rbTBI in young adulthood and behavioral and pathological outcomes were assessed at 10 and 15 months post-injury, respectively. This longitudinal approach was important for ensuring translational relevance since military service members are typically exposed to blast as young adults,^4^ yet faced an increased risk of developing dementia later in life.^49^ We hypothesized that rbTBI injured Tg12099 +/− rats would exhibit greater behavioral impairments, tau pathology, and glial activation compared with WT and sham controls.

## Materials and Methods

### Animals

**FIG. 1.**
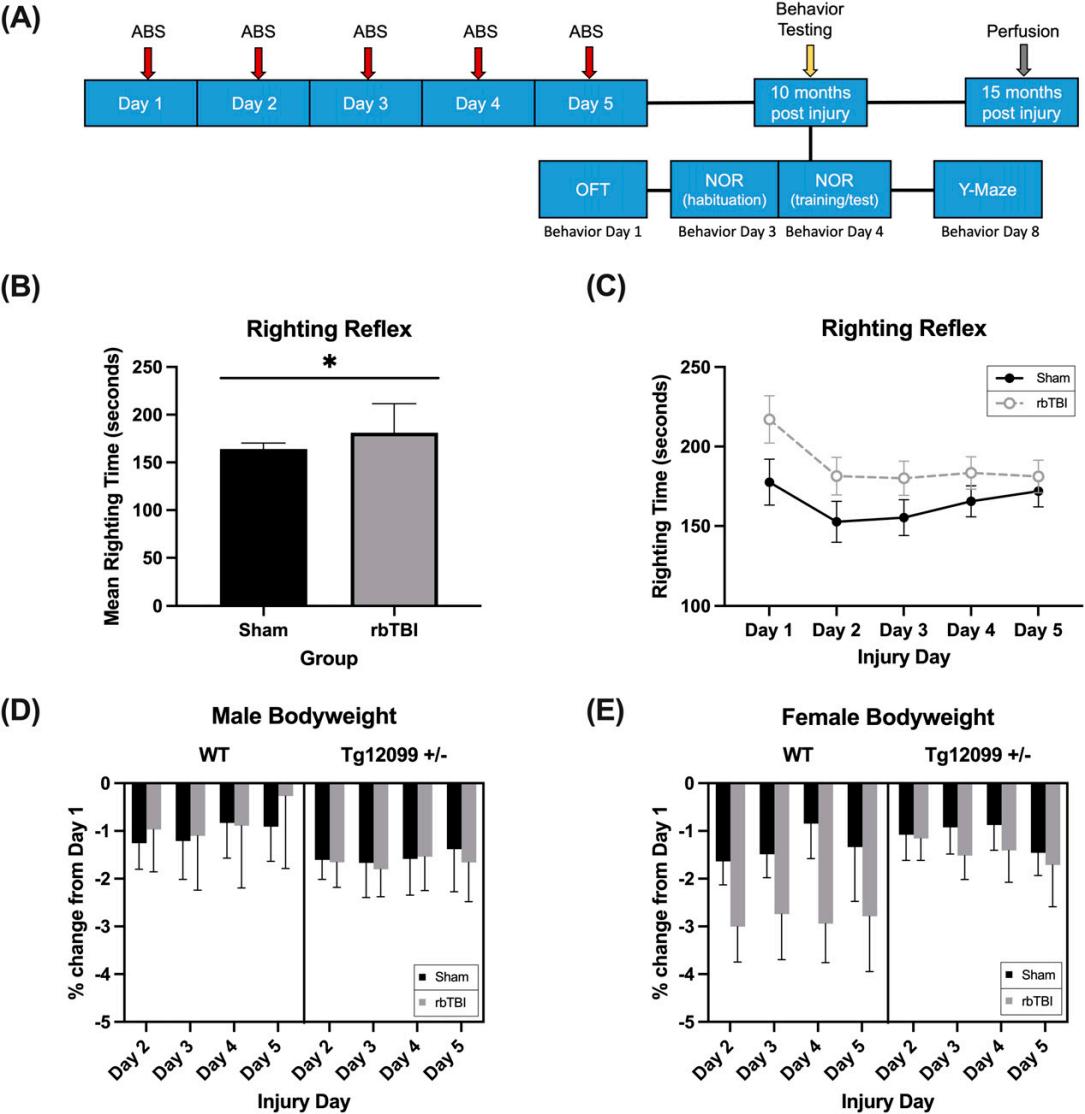
Experimental timeline, righting time, and body weight. **(A)** Schematic of the experimental procedure timeline. **(B)** Mean righting time was longer in blast-injured rats compared with sham rats (*p* = 0.032). **(C)** There was no effect of Day on righting time (*p* = 0.177). **(D–E)** Percent change in body weight from Day 1 (preblast) in male **(D)** and female **(E)** rats. All groups lost minimal weight from Day 1, but changes did not differ based on injury, genotype, or sex. *n* = 7–16 rats per group. *indicates *p* < 0.05. Data depict mean and SEM. ABS, Advanced Blast Simulator; rbTBI, repeated blast traumatic brain injury; SEM, standard error of the mean; Tg12099 +/−, transgenic heterozygous P301S Tau rat; WT, wild-type.

Animal experiments were conducted at the Uniformed Services University of the Health Sciences (USUHS): an AAALAC International accredited research facility. All procedures were approved by the USUHS Institutional Animal Care and Use Committee. A total of 89 2–3-month-old male (*n* = 49) and female (*n* = 40) Sprague Dawley rats were obtained from a breeding program between USUHS and UCSF at Charles River Laboratories (Wilmington, MA), including transgenic rats that were heterozygous for the mutated htau MAPT*P301S gene (Tg12099 +/−). For full characterization of Tg12099 +/− rats, refer to Ayers et al.^45^ The rats that did not have the transgene (labeled wild-type [WT]) were siblings of the Tg12099 rats. Genotype was determined using tail snips by real-time polymerase chain reaction (see Supplementary Table S1 in Ayers et al.^45^). Rats acclimated to the facilities for at least 3 days before experiments began and were housed in pairs (with the same genotype) when possible. Animals had access to food (Lab Diets, 5V75, 20% protein) and water *ad libitum* for the duration of the study. Rats were maintained on a standard 12 h:12 h light-dark cycle (lights on 06:00). All experimental procedures were performed during the light phase by female investigators. Animals were weighed once per week for the duration of the study. See Figure 1A for a graphical representation of overall experimental timeline.

### Advanced Blast Simulator

Male and female rats were randomly assigned to injured (rbTBI) or sham conditions. Injured animals were exposed to five blasts, at 24-h intervals, ∼19 psi peak pressure using the USUHS Advanced Blast Simulator (ABS) as previously described.^50^ Briefly, the ABS is comprised of a test section and driver section with pressure transducers placed within the inner wall to monitor incident pressure and shock wave velocity. A Valmex membrane (VALMEX® FR 1400 PVDF, Mehler-Texnologies, Martinsville, VA) separates the two sections. Rats were weighed immediately before each ABS or sham procedure, then individually anesthetized using an isoflurane induction chamber (4% isoflurane in 100% oxygen for 4–6 min). On the first injury day, all animals were injected with an implantable ID transponder (BMDS, IMI-500) while under anesthesia. Next, rats were quickly removed from the induction chamber and secured to a wooden tongue depressor using flexible gauze, to ensure there was minimal movement of the body during the blast exposure. The rat was then placed in the test section of the ABS and secured in a mesh holder in a prone position, with the head facing the direction of the oncoming blast wave. The test section door was then tightly sealed with the rat inside the ABS, compressed air accumulated in the driver section until the pressure was strong enough to burst the membrane, and the shock wave traveled down the ABS to where the animal was placed. An end wave eliminator reduced the reflection of the shock wave. After the blast wave exposure, the rat was quickly removed and placed in a clean cage on their back and assessed for the occurrence of apnea (there were no incidents of apnea) and to measure righting reflex; the duration of time required for the rat to return to a prone posture. Sham-treated rats were also anesthetized for 4–6 min and placed near the ABS but were not exposed to the blast wave. The righting reflex was recorded for sham rats in the same manner. Once rats regained consciousness, they were returned to their home cages and provided with acetaminophen (Tylenol) in their drinking water (7.5 mL Tylenol in 250 mL water) *ad libitum* for 24 h. Tylenol water was replaced with regular water 24 h after the final exposure.

### Behavior testing

Behavior testing began at 10 months post-injury (Fig. 1A). In the interim, rats were handled once per week when body weight was recorded, and by the experimenter once per day for 2 weeks before behavior testing began. Behavior testing of male rats occurred on separate days than female rats. Rats underwent the open field test (OFT) on Behavior Day 1. On Behavior Day 3, rats began the novel object recognition test (NOR). On Behavior Day 8, the Y-maze spontaneous alternation task (Y-maze) was administered. Before each test began, all rats to be tested were acclimated to the testing room in their home cage for 1 h.

### Open field test

A large open field arena (100W × 100L × 50H cm) made of gray PVC material (Total Plastics Int’l, Philadelphia, PA) was used for this test. Track lighting above the open field illuminated the arena to 50 lux. At the beginning of the test, the rat was placed in the bottom left corner of the arena, facing the wall, and allowed to freely explore for 10 min. A ceiling-mounted camera and Capture Star video recording software (CleverSys Inc., Reston, VA) recorded the animal’s movements throughout the duration of the test. Upon completion, the rat was returned to their home cage, and the arena was cleaned with a 70% EtOH solution and allowed to dry between subjects. Offline analysis was performed using Top Scan video tracking software (CleverSys Inc., Reston, VA), and provided measures of total distance traveled and time spent in center versus the perimeter of the arena. The center zone dimensions equaled 60 × 60 cm.

### Novel object recognition test

A medium-sized open field arena (70W × 70L × 50H cm) made of gray PVC material (Total Plastics Int’l, Philadelphia, PA) was used for this test. The open field arena was illuminated to 50 lux by overhead track lighting. The test began with a habitation phase, where the rat was placed in the bottom center of the empty arena facing a wall and allowed to freely explore for 10 min. Twenty-four hours later, the training phase began. Two identical objects were secured to the base of the arena floor using adhesive putty. The objects were placed in adjacent corners, 10 cm away from the wall, and 20 cm apart from each other. The rat was placed in the bottom center of the empty arena facing away from the objects. They were then allowed to freely explore for 5 min. The testing phase occurred after a 2-h delay. One object was replaced with a novel object. The rat was placed back in the same open field with one familiar object and one novel object and allowed to freely explore for 5 min. The corner in which the familiar object and novel object were placed was counterbalanced between test subjects and treatment groups. The arena and objects were cleaned with a 70% EtOH solution and allowed to dry between subjects. For all three phases, a ceiling-mounted camera and Capture Star video recording software (CleverSys Inc., Reston, VA) recorded the animal’s movements throughout the duration of each test. Offline analysis was performed using Top Scan video tracking software (CleverSys Inc., Reston, VA), including distance traveled and exploration time of each object. Object exploration was defined as the animal’s head being within 3 cm of the object. A preference index (PI) was calculated using the following formula: PI = 100 × [(novel object exploration duration)/(novel object exploration duration + familiar object exploration duration)]. PI scores range from 0 to 100%, where a score greater than 50% indicates more time spent with the novel object, a score less than 50% indicates more time spent with the familiar object, and a score of 50% indicates no preference. PI was also calculated during the training phase where PI = 100 × [(identical object 1 exploration duration)/(identical object 1 exploration duration + identical object 2 exploration duration)] to ensure the animals did not have a strong preference for one of the identical objects. In addition, % total object exploration was calculated as: total time exploring both objects/total time in arena. Animals that had <5% total object exploration time during either the training or test phase were excluded from the NOR analysis. This included four animals (1 male Tg12099 +/− Sham, 1 male Tg12099 +/− rbTBI, 1 male WT rbTBI, 1 female WT rbTBI). These four animals were included in all other behavioral analyses.

### Y-maze spontaneous alternation test

The Y-maze (MED-RAM-U-IR, Med Associates, Inc.) has three equally sized arms (4W × 18L × 7H in) each connected at a 120-degree angle with a triangular central zone. Visual cues were placed around the room and kept constant throughout the study. The test began by gently placing the animal in the bottom arm (start arm) of the Y-maze facing a wall. Animals were allowed to freely explore the Y-maze for 10 min. A ceiling-mounted camera connected to ANY-Maze software (Stoelting) recorded the animal’s movements. The maze was cleaned with a 70% EtOH solution and allowed to dry between subjects. This test measures spatial working memory by scoring the number of successful alternations the animal performs in the maze by visiting all three arms without going into the same arm twice in the three-arm sequence. Animals with an intact memory are expected to exhibit alternating behavior, meaning they visit all three arms in an alternating pattern, without returning to either of the arms that were explored most recently. An entry to an arm was counted when the center of the rat entered the arm. A successful alternation was counted when the animal entered three different arms consecutively. The percentage of correct alternations was calculated using the following formula: 100 × [(number of successful alterations)/(total number of entries − 2)].

### Histology

At 15 months post-injury (17–19 months of age), the rats were euthanized. The rationale for waiting 15 months after blast was to coordinate the timing utilized by Ayers et al.,^45^ when the greatest level of tau pathology had been reported. Rats were anesthetized with a mixture of ketamine and xylazine and transcardially perfused with chilled phosphate buffer solution and formalin solution. Brains were removed from the skull and further fixed in formalin solution for 24 h. They were then transferred to a 30% sucrose solution for 1 week before freezing and storing at −80°C. Brains were cut into 40 µm coronal sections using a Leica microtome and stored in cryoprotectant for at least 1 week before staining.

Free-floating sections were washed in 1× TBS-Triton (0.05%) three times to remove the cryoprotectant, followed by incubation with 0.3% hydrogen peroxide for 30 min. Sections were washed with TBS-Triton (0.05%) three times and then placed in blocking buffer (TBS-Triton (0.2%), goat serum, and 10% bovine serum albumin (BSA)) for 1 h at room temperature. The primary antibody was diluted in blocking buffer and applied to the sections for overnight incubation at 4°C. The primary antibodies were phospho-tau (Ser202, Thr205) (AT8) (1:500, ThermoFisher Scientific, MN1020); glial fibrillary acidic protein (GFAP) (1:500, ThermoFisher Scientific, MS-1376-P010); and ionized calcium-binding adaptor molecule 1 (Iba-1) (1:2000, Fujifilm Wako, 019-19741). The following day, the primary antibody solution was removed by washing sections with TBS-T (0.05%) three times. Secondary antibody was applied in blocking buffer (TBS-T with 0.05% triton, goat serum, and 10% BSA) for 1 h at room temperature. Secondary antibodies were goat anti-mouse (Jackson Immunoresearch, Code: 115-065-003, Lot: 155154) for AT8 (1:500) and GFAP (1:300) and goat anti-rabbit for Iba-1 (Jackson Immunoresearch, Code: 111-065-003, Lot: 155383 (1:300). Sections were then washed with TBS-T (0.05%) before incubation in ABC reagent (Vectastain ABC HRP Kit, PK-4000, Vector Laboratories) for 45 min at room temperature. The sections were washed again with TBS-T (0.05%) three times and then developed using the DAB Substrate Kit, Peroxidase (HRP), with Nickel, 3,3′-diaminobenzidine (SK-4100, Vector Laboratories). Developing times were kept consistent at 2 min for AT8 and 4 min for GFAP and IBA1. Sections were mounted on SuperFrost plus glass slides (Brain Research Laboratories, MA), and left to dry overnight. The next day, sections were dehydrated in ethanol gradients (75–100%), cleared in xylene twice, and cover slipped with Permount mounting media (Fisher Chemical).

### Imaging and analysis

Slides were scanned with a Zeiss AxioScan.Z1 (ZEISS, Oberkochen, Germany) at 10× magnification. Zen Blue 2.5 software (ZEISS, Oberkochen, Germany) was used to define regions of interest (ROIs). ROIs included: piriform cortex (PC), amygdala (Amy), CA3 region of the hippocampus (CA3), and paraventricular nucleus of the thalamus (PVT). To quantify ROIs, the percent area stained was measured in black/white images using the Yen threshold function on Image J software (NIH). Data were collected from three to four sections per animal, and the values were averaged. The imaging and quantification assessments were performed while blinded to the experimental condition. Please note for better visibility the brightness in the images in Figure 4 were adjusted, but no other changes were performed, and this change was after the images were quantified.

### Statistical analysis

Statistical analyses were performed using GraphPad Prism software (version 10; GraphPad Software, San Diego, CA), SPSS (version 21; IBM SPSS Statistics, Armonk, NY), and R Statistical Software (version 4.4.2, R Core team, 2024). Since the female and male rats were different body weights, and the time of isoflurane anesthesia varied to ensure rats were fully anesthetized, an analysis of covariance (ANCOVA) was performed for the duration of the righting reflex as genotype × injury × sex with body weight and isoflurane time as covariates. Preliminary tests indicated the analysis violated the assumption of Box’s Test of Equality of Covariance Matrices (F_105,5689.297_ = 1.304, *p* = 0.021), so significance was evaluated using the Greenhouse-Geiser correction for degrees of freedom for F-ratio testing. To analyze body weight by age, a quadratic regression model was compared with a linear regression model in R. For the behavioral tasks (OFT, NOR, and Y-maze), and GFAP and IBA-1 histology, three-way ANOVAs were performed (injury ×sex × genotype). Tukey’s test was used to correct for multiple comparisons. Effect sizes are reported for partial η^2^ computed in the SPSS program. Since preliminary tests indicated the measures for AT8 histology were not normally distributed, the non-parametric Kruskal–Wallis test was used, followed by Dunn’s multiple comparison test. For all tests, *p* < 0.05 was considered significant. Graphic presentation in the figures provides summaries based upon significant factor differences.

## Results

### Righting time, body weight

Righting time was assessed following isoflurane and shock wave exposure across each injury day and analyzed using a four-factor ANCOVA with body weight and isoflurane time as covariate factors (Supplementary Fig. S1). Controlling for body weight and isoflurane time, results indicated a non-significant main effect of sex (F_1, 71_ = 0.007, *p* = 0.934), suggesting no effect of sex on the time to regain consciousness after anesthesia and brain injury. However, a longer righting reflex duration was recorded in injured rats (F_1,71_ = 10.283, *p* = 0.002, η^2^ = 0.127) (Fig. 1B). Day was not a significant factor (F_2.826,200.633_ = 0.633, *p* = 0.177) (Fig. 1C).

A 4-factor ANOVA was performed to ascertain changes in body weight during the week of injuries. There were significant differences for the main effects for genotype (F_1,81_ = 6.349, *p* = 0.014, η^2^ = 0.073), injury (F_1,81_ = 11.775, *p* < 0.001, η^2^ = 0.127), and sex (F_1,81_ = 132.2 = 89.349, *p* < 0.001, η^2^ = 0.620), as well as differences over days (F_2.091,169.395_ = 17.890, *p* < 0.001, η^2^ = 0.181) (data not shown). The Tg12099 +/− rats weighed more than the WT rats, the rats exposed to rbTBI weighed less than the sham rats, and the males weighed greater than females. Although the rats were randomly assigned to the sham and rbTBI groups, a preliminary test indicated the rats differed in body weight before injuries (F_1,81_ = 9.631, *p* = 0.003, η^2^ = 0.106; rbTBI rats weighed less) and genotype (F_1,81_ = 5.674, *p* = 0.020, η^2^ = .065; WT rats weighed less) (data not shown). Therefore, measures of change from baseline body weights on Days 2–5 were computed as a percentage change in body weight. An injury × genotype × sex × day ANOVA indicated that on Days 2–5, the rats overall weighed less than on Day 1, but there were no significant differences in percent body weight loss over days or any of the fixed factors (Fig. 1D, E).

To analyze body weight by age across the duration of the study, a quadratic regression model was compared with a linear regression model in both male and female rats. The quadratic model was a statistically better fit with a lower AIC (*p* < 0.0001). A significant quadratic relationship was observed for all groups in both sexes (*p* < 0.0001). Males exhibited strong positive correlations between weight gain and age across all groups, with the strongest correlation found in the WT sham group (*r* = 0.9). The Tg12099 +/− rbTBI and WT rbTBI groups followed closely (*r* = 0.82 for both), and the Tg12099 +/− sham group had a similar correlation (*r* = 0.81) (Supplementary Fig. S2A). In females, the Tg12099 +/− sham group displayed the strongest correlation (*r* = 0.86), followed by the Tg12099 +/− rbTBI group (*r* = 0.69). Weaker moderate correlations were observed in the WT rbTBI and WT sham groups (*r* = 0.6 and 0.57, respectively) (Supplementary Fig. S2B). These findings suggest that while both sexes exhibit a quadratic weight gain or curved pattern over time, the strength of this relationship varies between groups and is generally stronger in males.

### Behavior testing

#### Open field test

**FIG. 2.**
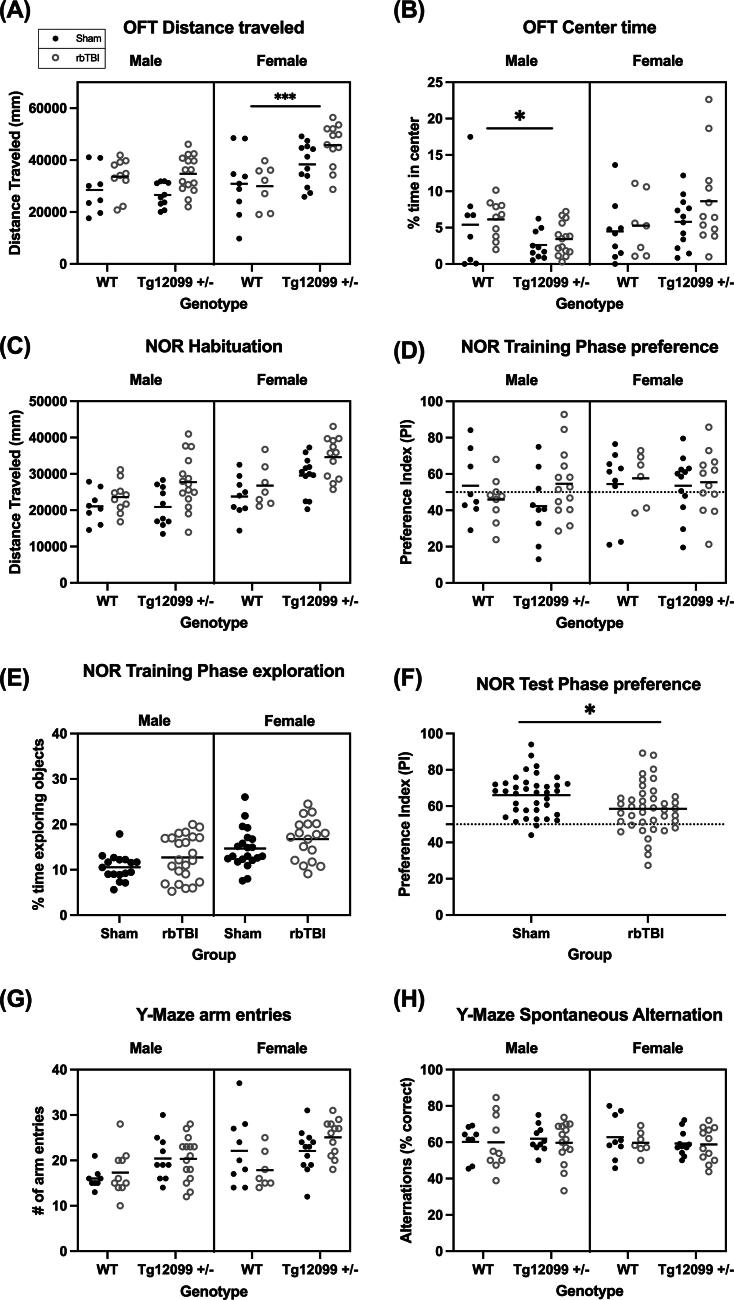
Behavior testing at 10 months post-injury. **(A)** OFT distance traveled. Blast-injured animals traveled a greater distance compared with sham animals. There was also a main effect of sex (F > M) and genotype (Tg12099 +/− > WT). In addition, there was a significant interaction between genotype × sex. Tg12099 +/− females traveled farther than WT females, while activity levels did not differ by genotype in males. **(B)** OFT center time. There was a significant interaction between genotype and sex. Tg12099 +/− males spent less time in the center than WT males, while center time did not differ by genotype in females. **(C)** NOR habituation. There were main effects of injury, sex, and genotype where activity levels were increased in blast-injured animals compared with sham, females traveled greater distances than males, and Tg12099 +/− rats traveled greater distances than WT rats. **(D)** NOR training phase preference. No differences were seen in object preference when exploring two identical objects during the training phase. The dashed line depicts 50% PI indicating no preference. **(E)** NOR training phase exploration. Injured animals spent more time exploring the identical objects compared with sham (*p* = 0.0127) and females spent more time exploring the identical objects than males (*p* = 0.0001). **(F)** NOR test phase. Injured animals had a lower PI than sham animals, indicating less preference for the novel object (*p* = 0.0108). **(G)** Y-maze arm entries. Females had a greater number of arm entries compared with males (*p* = 0.0015), and Tg12099 +/− rats had a greater number of arm entries compared with WT rats (*p* = 0.0042). **(H)** Y-maze spontaneous alternation. No significant differences were observed for % correct alternation. Lines depict means (*n* = 7–16 rats per group). *p < 0.05, ***p < 0.001. Data were combined for presentation purposes in (E) (no genotype effect) and (F) (no genotype or sex effect). NOR, novel object recognition; OFT, open field test; PI, preference index; rbTBI, repeated blast traumatic brain injury.

A three-way ANOVA was used to analyze distance traveled in the open field (Fig. 2A). There was a main effect of injury (F_1, 75_ = 7.069, *p* = 0.0096, η^2^ = 0.086), where activity levels were increased in blast-injured animals compared with sham rats. There was also a main effect of sex (F_1, 75_ = 8.297, *p* = 0. 0052, η^2^ = 0.100), where females traveled greater distances than males, and a main effect of genotype (F_1, 75_ = 9.153, *p* = 0.0034, *p* = 0.003, η^2^ = 0.109), where Tg12099 +/− rats traveled a greater distance compared with WT rats. In addition, there was a significant genotype × sex interaction (F_1, 75_ = 10.49, *p* = 0.0018, η^2^ = 0.230), where female Tg12099 +/− rats exhibited significantly greater travel distances than female WT rats, while male activity levels did not differ by genotype. The percentage of time spent in the center of the arena was analyzed as a measure of anxiety-like behavior (Fig. 2B). A three-way ANOVA revealed a significant interaction between genotype and sex (F_1,75_ = 7.742, *p* = 0.0068, η^2^ = 0.094). Center time percentage was lower in male Tg12099 +/− rats when compared with male WT rats, yet there was no genotype difference in female rats.

#### Novel object recognition

Distance traveled during the habituation phase of the NOR test was analyzed using a three-way ANOVA (Fig. 2C). Results indicated significant main effects of injury (F_1,75_ = 11.31, *p* = 0.0012, η^2^ = 0.131), sex (F_1,75_ = 17.13, *p* < 0.0001, η^2^ = 0.186), and genotype (F_1,75_ = 11.62, *p* = 0.0011, η^2^ = 0.134). Blast-injured animals exhibited increased activity levels compared with sham controls. Females traveled greater distances than males, and Tg12099 +/− rats traveled greater distances than WT rats.

The training phase of NOR did not reveal any significant differences in PI, suggesting that the animals had no preference for either of the identical objects (Fig. 2D). Percentage of time spent exploring both objects during this phase was also analyzed using a three-way ANOVA and significant main effects for injury (F_1,71_ = 6.537, *p* = 0.0127, η^2^ = 0.083) and sex were found (F_1,71_ = 16.74, *p* = 0.0001, η^2^ = 0.190) (Fig. 2E). Compared with males, females explored both objects for a longer duration and injured animals spent more time exploring both objects than sham animals.

During the test phase, a PI was calculated and a three-way ANOVA revealed a main effect of injury (F_1,71_ = 6.857, *p* = 0.0108, η^2^ = 0.088), where injured animals had a lower PI than sham animals, indicating less preference for the novel object (Fig. 2F**).**

#### Y-maze spontaneous alternation task

The total number of arm entries on the Y-maze was analyzed with a three-way ANOVA and main effects of genotype and sex were found (F_1,75_ = 10.90, *p* = 0.0015, η^2^ = 0.127, F_1,75_ = 8.74, *p* = 0.0042, η^2^ = 0.104, respectively) where Tg12099 +/− rats had a greater number of arm entries than WT rats, and females had more arm entries than males (Fig. 2G). However, a three-way ANOVA revealed no significant differences in spontaneous alternation on the Y-maze, suggesting working spatial memory was not altered by injury, genotype, or sex (Fig. 2H).

### Histology

#### AT8 phosphorylated tau

**FIG. 3.**
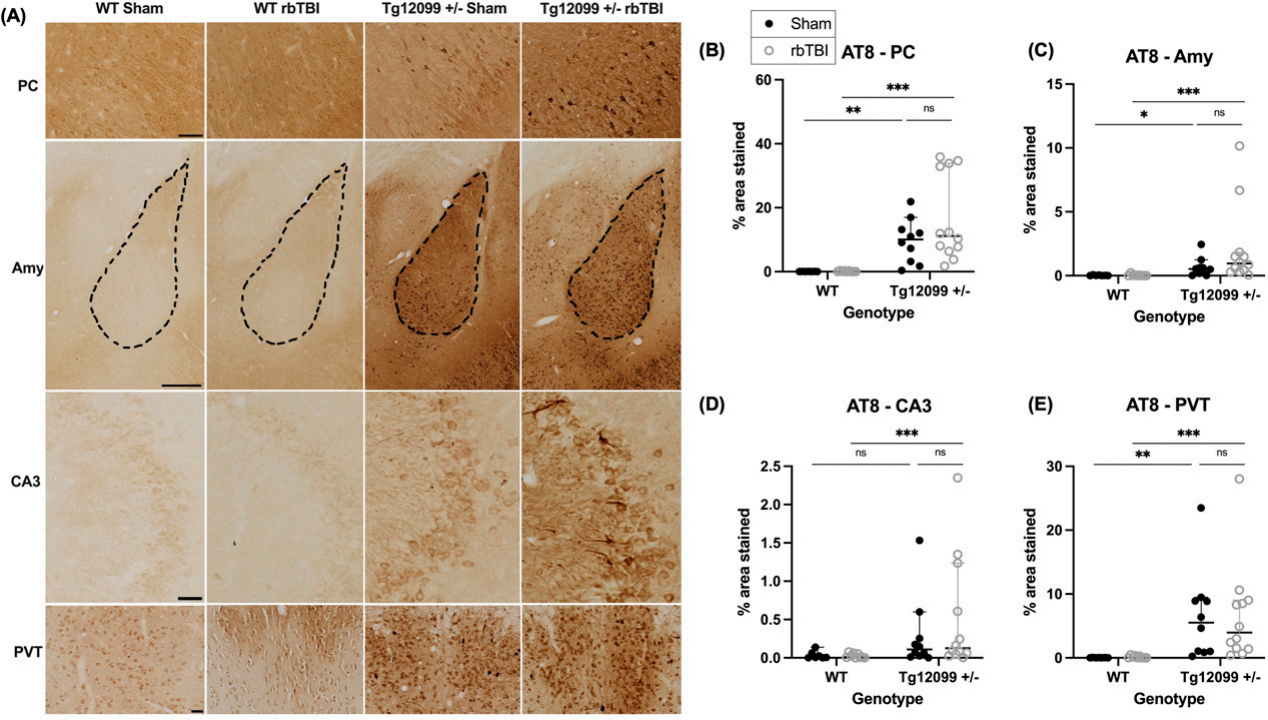
Immunohistochemistry for AT8 (phosphorylated tau) at 15 months post-injury (17–19 months old). **(A)** Representative images of piriform cortex (PC), amygdala (Amy), cornu ammonis 3 region of the hippocampus (CA3), and paraventricular nucleus of the thalamus (PVT). Scale bars = 100 µm for PC, CA3, and PVT; scale bar = 500 µm for Amy. **(B–E)** Quantification of percentage of area stained in the PC, Amy, CA3, and PVT. Both injured and sham Tg12099 +/− rats exhibited greater AT8 staining compared with their respective WT controls in the PC, Amy, and PVT. In CA3, only injured Tg12099 +/− rats exhibited greater AT8 staining compared with injured WT rats **(D)**. The Kruskal—Wallis rank test, followed by Dunn’s multiple comparison test, was used to analyze each region. There were no sex differences so male and female data were pooled for analysis. Lines represent median and error bars indicate 95% CI (*n* = 7–12 rats per group); ns = not significant, *p < 0.05, **p < 0.01, ***p < 0.001. AT8, phosphorylated tau (Serine 202/Threonine 205).

AT8 immunohistochemistry revealed increased tau phosphorylation in several brain regions of the Tg12099 +/− rats compared with WT controls, regardless of injury status. Representative images of AT8 staining are shown in Figure 3A. Given the non-normal distribution of data, the Kruskal–Wallis test followed by Dunn’s multiple comparison test was employed to analyze each ROI. By graphical inspection there were no sex-based differences in AT8 staining, so data from male and female animals were pooled for analysis. In the PC, significant group differences in AT8-positive percentage area stained were observed (*H* = 27.56, *p* < 0.0001). *Post hoc* analysis indicated that both injured and sham Tg12099 +/− rats exhibited higher AT8 staining compared with their respective WT controls (*p* = 0.0003 and *p* = 0.0028, respectively) (Fig. 3B). A similar pattern was evident in the amygdala (Amy) (*H* = 23.24, *p* < 0.0001), with increased AT8 staining in both injured and sham Tg12099 +/− rats relative to WT controls (*p* = 0.0002 and *p* = 0.0456, respectively) (Fig. 3C). In the CA3 region of the hippocampus (CA3), significant group differences in the AT8-positive percentage area stained were observed (*H* = 12.96, *p* = 0.0047). *Post hoc* analysis indicated that injured Tg12099 +/− rats exhibited higher AT8 staining compared with injured WT rats (*p* = 0.0265) (Fig. 3D**).** Finally, significant group differences in the AT8-postive percentage area stained were observed in the PVT (*H* = 26.14, *p* < 0.0001). *Post hoc* analysis indicated that both injured and sham Tg12099 +/− rats exhibited higher AT8 staining compared with their respective WT controls (*p* = 0.0013 and *p* = 0.0012, respectively) (Fig. 3E).

#### Glial fibrillary acidic protein

**FIG. 4.**
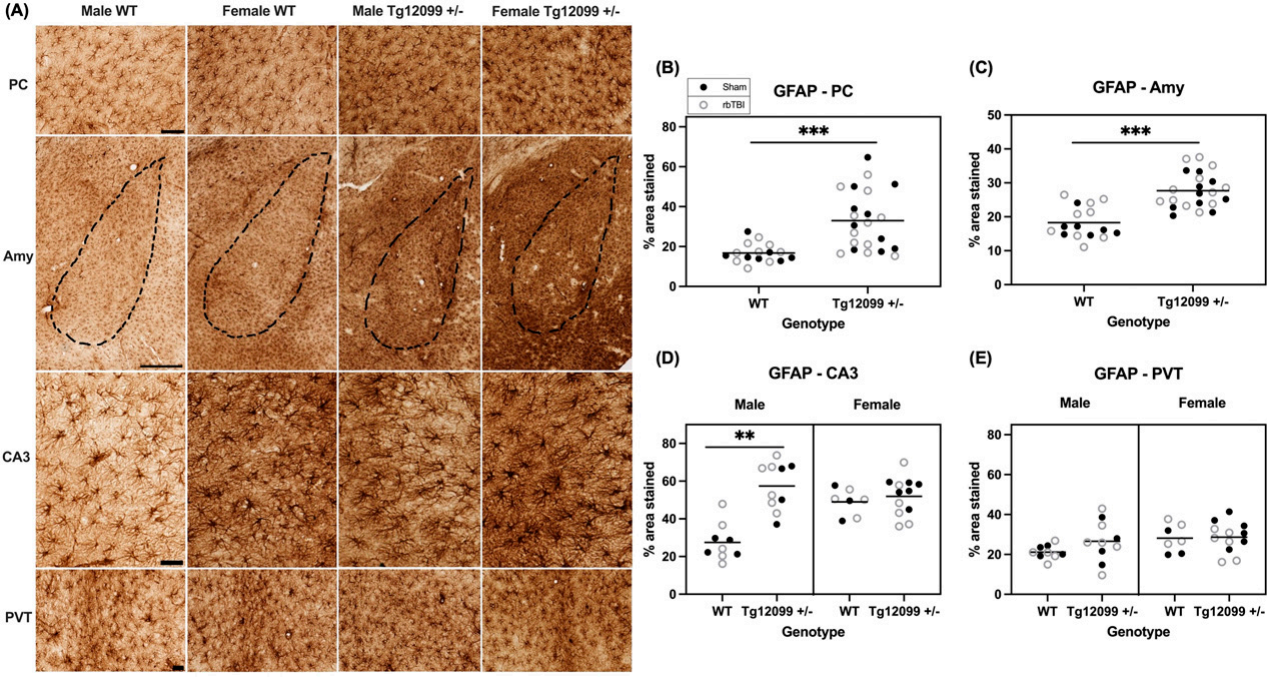
Immunohistochemistry for GFAP at 15 months post-injury (17–19 months old). **(A)** There were no injury differences in any brain region so representative images depict both sex and genotype in the piriform cortex (PC), amygdala (Amy), cornu ammonis 3 region of the hippocampus (CA3), and paraventricular nucleus of the thalamus (PVT). Scale bars = 100 µm for PC, CA3, and PVT; scale bar = 500 µm for Amy. **(B–D)** Quantification of percentage of area stained in the PC, Amy, CA3, and PVT. Tg12099 +/− rats exhibited higher GFAP-positive area stained compared with WT rats in the PC and Amy. In CA3, male Tg12099 +/− rats had higher GFAP than male WT rats, but in female rats both WT and Tg12099 +/− GFAP levels were higher than male WT. **(D)** No differences were seen in astrogliosis in the PVT. A three-way ANOVA was used to analyze each region. Lines indicate mean (*n* = 7–12 rats per group). Data were combined for presentation purposes in (B) and (C) (no injury or sex effect), (D) and (E) (no injury effect). GFAP, glial fibrillary acidic protein.

GFAP immunohistochemistry revealed increased astrogliosis in Tg12099 +/− rats. Representative images of GFAP staining are shown in Figure 4A. Each ROI was analyzed to assess for differences in GFAP-positive percentage area stained using a three-way ANOVA. Results indicated a significant main effect of genotype (F_1,30_ = 15.31, *p* = 0.0005, η^2^ = 0.338), with Tg12099 +/− rats displaying elevated GFAP levels in the PC compared with WT controls (Fig. 4B). Similarly, in the amygdala Tg12099 +/− rats had greater GFAP staining compared with WT rats (F_1, 30_ = 28.38, *p* < 0.0001, η^2^ = 0.481) (Fig. 4C). In the CA3 region, a significant main effect of genotype was also found (F_1, 30_ = 20.94, *p* < 0.0001, η^2^ = 0.411). However, in this region, a sex main effect (F_1, 30_ = 5.362, *p* = 0.0276, η^2^ = 0.152) and a significant sex × genotype interaction were also identified (F_1, 30_ = 13.99, *p* = 0.0008, η^2^ = 0.318). Overall, female rats demonstrated greater GFAP levels than males, and while WT males exhibited lower astrogliosis in the CA3 region compared with Tg12099 +/− males (*p* < 0.001), GFAP staining did not differ by genotype in female rats (*p* = 0.564, Fig. 4D). Finally, there were no significant differences found in GFAP levels in the PVT (Fig. 4E).

#### Ionized calcium-binding adaptor molecule 1

**FIG. 5.**
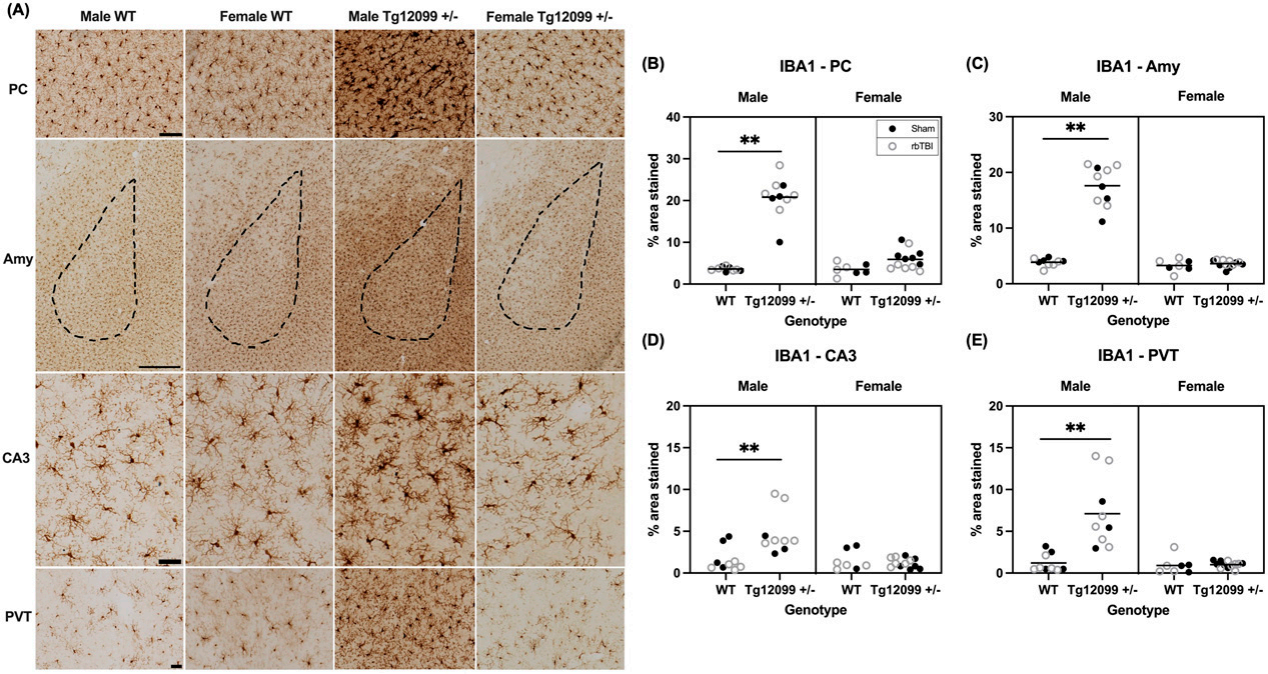
Immunohistochemistry for IBA1 at 15 months post-injury (17–19 months old). **(A)** Representative images by sex and genotype in the piriform cortex (PC), amygdala (Amy), CA3 region of the hippocampus, and paraventricular nucleus of the thalamus (PVT). Scale bars = 100 µm for PC, CA3, and PVT; scale bar = 500 µm for Amy. **(B–E)** Quantification of percentage area stained in the PC, Amy, CA3, and PVT. Male Tg12099 +/− rats exhibited higher IBA1-positive area stained compared with male WT rats and both female groups in all four brain regions. In the CA3 region, there was also an injury and genotype interaction, where injured Tg12099 +/− rats had higher levels of IBA1 compared with injured WT rats, but there was no genotype difference in sham rats **(D)**. A three-way ANOVA was used to analyze each region. Lines indicate mean (*n* = 3–12 rats per group). Data were combined for presentation purposes in (no injury effect). IBA1, ionized calcium-binding adaptor molecule 1.

IBA1 immunohistochemistry revealed increased microgliosis in male Tg12099 +/− rats. Representative images of IBA1 staining are shown in Figure 5A. A three-way ANOVA was conducted to assess the IBA1-positive percentage area stained for each ROI. In the PC, there were main effects of genotype and sex, (F_1, 30_ = 105.7, *p* < 0.0001, η^2^ = 0.779, and F_1,30_ = 61.53, *p* < 0.0001, η^2^ = 0.672, respectively). There was also a significant genotype × sex interaction (F_1,30_ = 59.46, *p* < 0.0001, η^2^ = 0.665), where Tg12099 +/− rats exhibited increased IBA1 in the PC compared with females. No such sex difference was observed in WT rats (Fig. 5B). Similarly, in the amygdala, main effects of genotype and sex were found (F_1,30_ =110.1, *p* < 0.0001, η^2^ = 0.786 and F_1, 30_ = 119.6, *p* < 0.0001, η^2^ = 0.799, respectively). Again, there was a significant genotype × sex interaction (F_1,30_ = 99.79, *p* < 0.0001, η^2^ = 0.769) in the same pattern as the PC (Fig. 5C). In CA3 region of the hippocampus, there were main effects of genotype and sex (F_1,29_ =5.684, *p* = 0. 0239, η^2^ = 0.164 and F_1,29_ = 11.02, *p* = 0.0024, η^2^ = 0.275, respectively). There was also a significant genotype x sex interaction (F_1,29_ = 9.613, *p* = 0.0043, η^2^ = 0.249) in the same pattern as the PC and amygdala. Additionally, a significant genotype x injury interaction was found, where injured Tg12099 +/− rats had greater positive-IBA1 percentage area stained compared with injured WT rats, but there were no genotype differences in sham rats (F_1,29_ = 8.521, *p* = 0.0067, η^2^ = 0.227, Fig. 5D**).** Lastly, in the PVT main effects of sex and genotype were found (F_1,29_ =13.61, *p* = 0.0009, η^2^ = 0.319 and F_1,29_ = 16.11, *p* = 0.0004, η^2^ = 0.357, respectively), as well as a sex × genotype interaction (F_1,29_ =12.30, *p* = 0.0015, η^2^ = 0.298, Fig. 5E**).** Notably, two of the male Tg12099 +/− (1 sham, 1 rbTBI) rats displayed outlier areas of IBA1 “clusters” and are shown in Supplementary Figure S3. If the cluster was located in a ROI, then that data point was removed from the analysis above as it heavily skewed the data distribution.

## Discussion

Contrary to our hypothesis, the current study did not find evidence that repeated blast injury in young adulthood worsens outcomes in tauopathy-prone rats. However, notable changes across several measurements were observed that are likely attributable to blast exposure, genotype, and/or sex.

In animal models, the righting reflex, a reflexive action originating in the brainstem, is often used as a tool for assessing the impact of anesthetics^51–53^ and brain injury.^54,55^ Specifically, the loss of righting reflex is a proxy for loss of consciousness in pre-clinical models of TBI.^56^ In the present study, after controlling for bodyweight and isoflurane time, injured animals took significantly longer to right themselves compared with sham animals, indicating a concussive effect of the blast exposure.

Given the longitudinal design of the study, health problems were recorded and are reported in Supplementary Table S1 for transparency and awareness for future studies using Tg12099 rats. Notably, malocclusion was common in Tg12099 +/− rats, occurring in ∼35% of the Tg12099 rats, compared with ∼11% WT rats. Malocclusion, characterized by the misalignment of the upper and lower incisors leading to excessive incisor growth, has been previously shown to result from different environmental and genetic factors.^57,58^ A study of Wistar rats reported the incidence of malocclusion to reach 1% across 2 years of age.^59^ This incidence appears to increase with age, as another study following Wistar rats found incidence of 32.5% by age 3, with the majority of cases occurring between 23 and 36 months of age.^60^ To our knowledge, the incidence of malocclusion has not been reported in either WT or transgenic Sprague Dawley rats. A comparison of the incidence of malocclusion in the WT and Tg12099 +/− rats suggests the groups do not represent equivalent distributions (Fisher’s exact test, percentage of malocclusions in WT and Tg12099 +/− rats, *p* < 0.0126). Careful attention should be paid to this condition, especially in aging studies, as undetected or untreated cases can lead to eating difficulties, tissue damage, stress, and even death.^57,58,60^ In this study, animals presenting with malocclusion had their teeth monitored and trimmed as needed to avoid these complications. To further monitor the health of the animals in this long-term study, body weight measurements were taken weekly and are reported in Supplementary Figure S2. The trajectories of weight gain as the groups aged were similar, and there was no evidence of a significant diminution of body weight in the Tg12099 +/− rats as they aged, compared with the WT animals.

The present study assessed behavioral outcomes at 10 months following rbTBI with the hypothesis that blast-injured Tg12099 +/− rats would perform worse compared with Tg12099 +/− sham rats and WT rats. While this result was not seen on any of the behavioral tests, there were persistent effects of blast exposure. On the OFT, blast-injured rats traveled a greater distance compared with sham rats. This aligns with clinical observations of post-TBI hyperactivity, which may manifest as impulsivity, agitation, restlessness, and disinhibition,^61–63^ although these behaviors were not assessed in the present study. In previous studies, increased activity levels have been demonstrated on the OFT in animals with impact TBI,^64,65^ as well as after repeated blast exposure.^66^ However, other studies have reported either no differences^23,67–70^ or reduced activity levels after blast exposure on this test.^24,71–75^ These mixed findings may be due to different blast devices, time post-injury, and OFT duration. To our knowledge, this is the first study to demonstrate greater travel distances on the OFT at 10 months post rbTBI. In addition, Tg12099 +/− females traveled farther than WT females on the OFT, while activity levels did not differ by genotype in males, highlighting the sexual dimorphism of this behavior in Tg12099 rats. This same characteristic of greater activity levels has been demonstrated in a study of P301S female mice.^76^ Additionally, we assessed time spent in the center of the open field. While no genotype differences were observed in females, possibly due to their higher activity levels, Tg12099 +/− males exhibited significantly less center time than WT males, suggesting an anxiety-like phenotype in Tg12099 +/− males.

During the habituation phase of NOR, where animals were placed in a smaller open arena compared with the OFT, the injured animals again traveled a greater distance compared with sham animals, further validating a long-lasting phenomenon of greater activity levels in the blast-injured animals as was seen on the OFT. In the testing phase of NOR, blast-injured rats displayed impaired recognition memory, as evidenced by a lower PI compared with sham controls. These findings align with previous studies reporting deficits on NOR at various time points, ranging from 7 days up to 3 months post-injury.^77–83^ Further, one study reported deficits on NOR at 5 months after rbTBI, but in that study the training and testing phase were separated by 4 weeks, rather than 2 h as in the present study.^23^ Another study assessed blast-exposed rats on the NOR test at 7 months post-injury and found a reduction in overall exploratory behavior, as well as subtle deficits in novel object exploration.^24^ The present study suggests that these cognitive deficits in recognition memory persist up to 10 months after rbTBI. Given the critical role of the entorhinal, perirhinal, and parahippocampal cortices on NOR performance,^84^ these findings suggest these regions may be particularly vulnerable to the long-term effects of blast injury. Finally, there were no deficits on the Y-maze spontaneous alternation task, suggesting spatial working memory may remain intact in Tg12099 +/− rats regardless of blast exposure. The lack of observed cognitive deficits in Tg12099 +/− rats may indicate that heterozygosity for the mutated P301S gene, while potentially contributing to subtle neuropathological changes, may not be sufficient to induce significant cognitive impairments, even when these animals are subjected to blast overpressure.

Since abnormally phosphorylated tau is a hallmark of tauopathy, we investigated whether blast injury in young adulthood would increase phosphorylated tau in Tg12099 +/− rats when assessed at 15 months post-injury. Using the marker AT8, which detects phosphorylated tau at Serine 202 and Threonine 205 residues, we found that blast did not worsen tau pathology in Tg12099 +/− rats. While there were increases in phosphorylated tau in the Tg12099 +/− rats compared with WT rats, these differences were apparent in both sham and blast-injured rats in the PC, amygdala, and PVT. In CA3, only injured Tg12099 +/− rats exhibited higher AT8 staining compared with injured WT rats. We hypothesized that we would see increases in blast-injured Tg12099 +/− rats compared with sham Tg12099 +/− rats, however, that did not occur in the present study suggesting our model of repeated blast exposure does not worsen tau pathology in Tg12099 +/− rats. It is also possible that the specific parameters of the blast paradigm used in this study may not have been severe enough to induce significant exacerbation of tau pathology in this model. Furthermore, future studies are needed utilizing additional timepoints to determine the progression of tau pathology within the Tg12099 +/− model.

Given the complex interplay between glial activation and tauopathy, we also investigated whether there was gliosis at 15 months post-injury. Since activated astrocytes and microglia are pathological hallmarks of tauopathy,^85^ we stained for GFAP and IBA1, respectively. We observed increases in the percentage area stained of GFAP in Tg12099 +/− rats compared with WT rats in the PC and amygdala. While in CA3, male Tg12099 +/− rats had higher GFAP than male WT rats, in female rats both WT and Tg12099 +/− had greater GFAP levels than male WT rats. However, we did not observe any blast-related effects on astrogliosis. We also stained for IBA1 to assess microgliosis and found male Tg12099 +/− rats exhibited higher IBA1-positive area stained compared with male WT rats and both female groups in all four brain regions. Once again, there was no injury-related effect. While increases in IBA1 and GFAP have been reported in transgenic tau rats,^45,86,87^ none have evaluated sex differences. These sex differences in glial activation are particularly interesting given that we did not see any sex differences in AT8 accumulation in Tg12099 +/− rats. Notably, two of the male Tg12099 +/− rats (1 sham, 1 rbTBI) displayed outlier areas of IBA1 “clusters,” which was a unique staining pattern that we have not seen reported in similar models of blast injury or tauopathy (Supplementary Fig. S3).

In conclusion, our hypothesis that repeated blast injury in young adulthood will worsen outcomes in Tg12099 +/− rats was not supported. Yet, our results suggest the permanency of deficits from repeated blast exposure, including increased activity levels and cognitive deficits at 10 months post-injury. In addition, this study provides the first behavioral phenotype, although limited, of the Tg12099 +/− model. Further, repeated blast exposure did not increase pathology in rats predisposed to tauopathy, which suggests the lasting effects of blast exposure may not be related to tau. This notion aligns with a recent clinical study evaluating the brains from 225 deceased service members where there was only evidence of tauopathy in 10 (4.4%) of the brains. Only 3 out of the 10 had a history of blast exposure, but importantly all 10 had a history of contact sports, suggesting impact TBI, rather than blast exposure, is the primary risk factor in tauopathy.^88^ The Tg12099 rat is a unique model and can be used in future studies to investigate whether other injury mechanisms, such as impact TBI, may trigger tauopathy in genetically predisposed rats.

## Data Availability

The data are available at odc-tbi.org

## Abbreviations Used

ABS: Advanced Blast Simulator
Amy: Amygdala
ANCOVA: Analysis of covariance
ANOVA: Analysis of variance
AT8: Phosphorylated tau (Serine 202, Threonine 205)
BSA: Bovine serum albumin
CA3: CA3 region of the hippocampus
GFAP: Glial fibrillary acidic protein
hTau: Human tau
Iba-1: Ionized calcium-binding adaptor molecule 1
IEDs: Improvised explosive devices
MAPT: microtubule-associated protein tau
NOR: Novel object recognition test
OFT: Open field test
PC: Piriform cortex
PET: positron emission tomography
PI: Preference index
PVT: Paraventricular nucleus of the thalamus
rbTBI: Repeated blast traumatic injury
ROIs: Regions of interest
SEM: Standard error of the mean
TBI: Traumatic brain injury
TBS: Tris buffered saline
Tg12099: Mutated P301S human tau gene
USUHS: Uniformed Services University of the Health Sciences
VALMEX: Valmex membrane
WT: Wild type
