# Supplementary material for: Chronic Neurobehavioral and Neuropathological Consequences of Repeated Blast Exposure in P301S Transgenic Tau Rats
Source: Neurotrauma Rep. 2025 Apr 29;6(1):374–90. doi: 10.1089/neur.2024.0168 (PMC12281117; doi:10.1089/neur.2024.0168)
Supplement: Supplementary Table S1 [file neur.2024.0168_supplementary_table_s1.docx]

| **Supplemental Table 1.** Health characteristics of rats through 17-19 months of age.^[[1]](#footnote-1)^ | | | | | | | | | |
| --- | --- | --- | --- | --- | --- | --- | --- | --- | --- |
|  | **Male** | | | | **Female** | | | | |
| **Health Problem** | **WT Sham (n=11)** | **WT rbTBI**  **(n=10)** | **Tg12099 +/- Sham**  **(n=12)** | **Tg12099 +/- rbTBI**  **(n=16)** | **WT Sham**  **(n=9)** | **WT rbTBI**  **(n=7)** | **Tg12099 +/- Sham**  **(n=12)** | **Tg12099 +/- rbTBI**  **(n=12)** |  |
| **Tumor – no. (%)** | 0(0) | 0(0) | 0(0) | 1(6) | 0(0) | 0(0) | 0(0) | 0(0) |  |
| **Wound/Infection/ Skin Ulcer – no. (%)** | 0(0) | 0(0) | 2(16) | 1(6) | 0(0) | 0(0) | 1(8) | 0(0) |  |
| **Malocclusion – no. (%)** | 2(18) | 0(0) | 5(41) | 6(38) | 1(11) | 1(14) | 4(33) | 3(25) |  |
| **Paralysis – no. (%)** | 2(18) | 0(0) | 0(0) | 1(8) | 0(0) | 0(0) | 1(8) | 0(0) |  |

1. Health problems were detected by veterinary staff and lab investigators. All animals included in the behavioral analysis were healthy at the time of testing. If the health problem occurred prior to the behavior testing time point, the animal was not included in the behavior testing. [↑](#footnote-ref-1)
