# Supplementary material for: Chronic Neurobehavioral and Neuropathological Consequences of Repeated Blast Exposure in P301S Transgenic Tau Rats
Source: Neurotrauma Rep. 2025 Apr 29;6(1):374–90. doi: 10.1089/neur.2024.0168 (PMC12281117; doi:10.1089/neur.2024.0168)
Supplement: Supplementary Figure S1 [file neur.2024.0168_supplementary_figure_s1.docx]

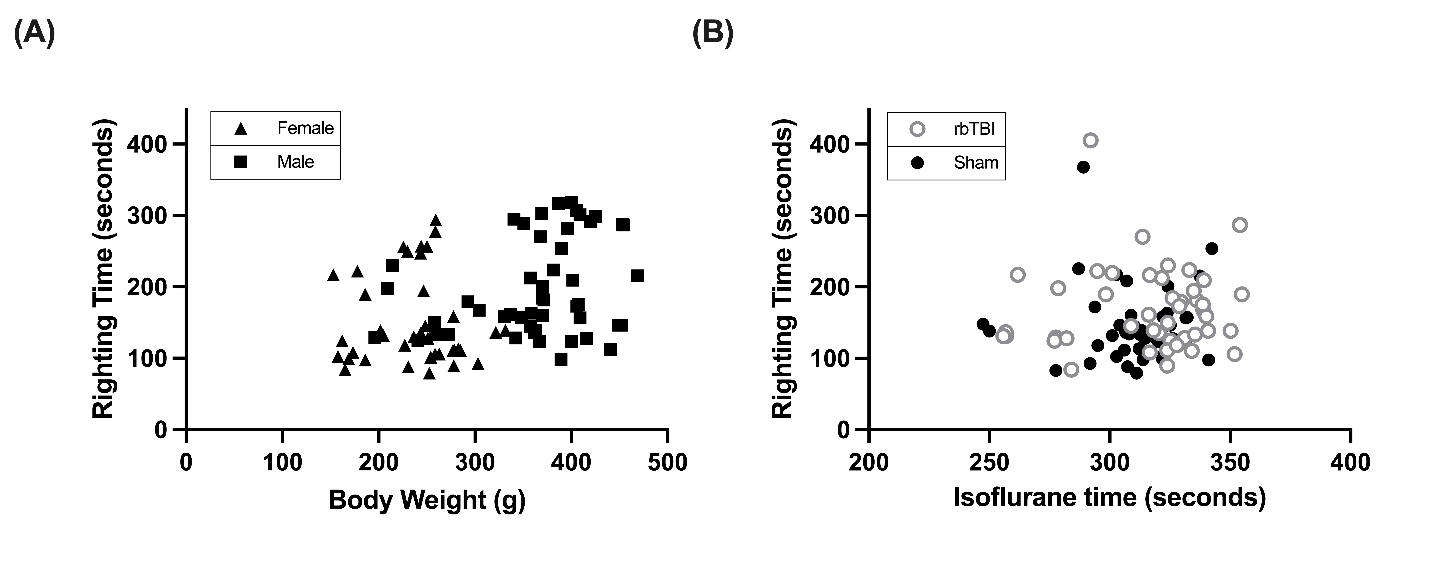


***Supplemental Figure 1.*** **(A) Two-dimensional scatter plot of initial** body weight and average righting time across all five injury days. (B) Scatter plot of average isoflurane exposure time and average righting time across all five injury days. **Note:** Both body weight and isoflurane exposure time were controlled for as covariate factors in the statistical analyses presented in Figures 1B and 1C. rbTBI, repeated blast traumatic brain injury.
