# Supplementary material for: Chronic Neurobehavioral and Neuropathological Consequences of Repeated Blast Exposure in P301S Transgenic Tau Rats
Source: Neurotrauma Rep. 2025 Apr 29;6(1):374–90. doi: 10.1089/neur.2024.0168 (PMC12281117; doi:10.1089/neur.2024.0168)
Supplement: Supplementary Figure S2 [file neur.2024.0168_supplementary_figure_s2.docx]

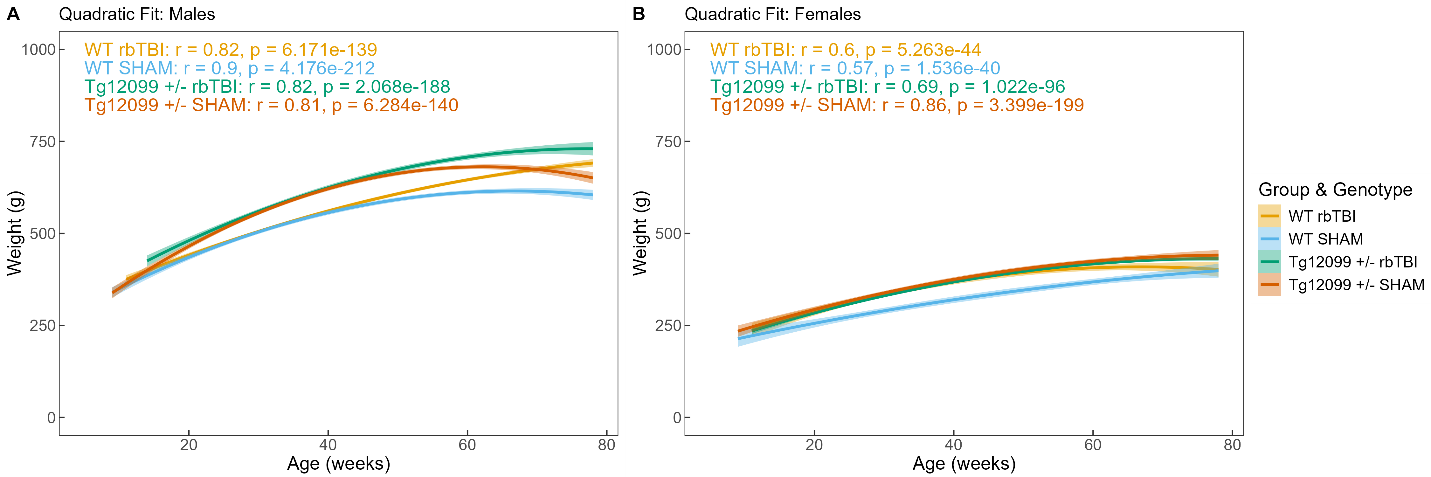


***Supplemental Figure 2.*** A significant quadratic relationship was observed for all groups in both sexes (p < .0001). Males are shown in A, Females are shown in B. There were strong positive correlations between weight gain and age across all groups. The strength of this relationship varies between groups as depicted by r values shown in legend. *n* = 7-16 rats per group. Data depicts mean and SEM. rbTBI, repeated blast traumatic brain injury. WT, wild-type. Tg12099 +/-, transgenic heterozygous P301S Tau rat. SEM, Standard error of the mean.
