# Supplementary material for: Chronic Neurobehavioral and Neuropathological Consequences of Repeated Blast Exposure in P301S Transgenic Tau Rats
Source: Neurotrauma Rep. 2025 Apr 29;6(1):374–90. doi: 10.1089/neur.2024.0168 (PMC12281117; doi:10.1089/neur.2024.0168)
Supplement: Supplementary Figure S3 [file neur.2024.0168_supplementary_figure_s3.docx]

***
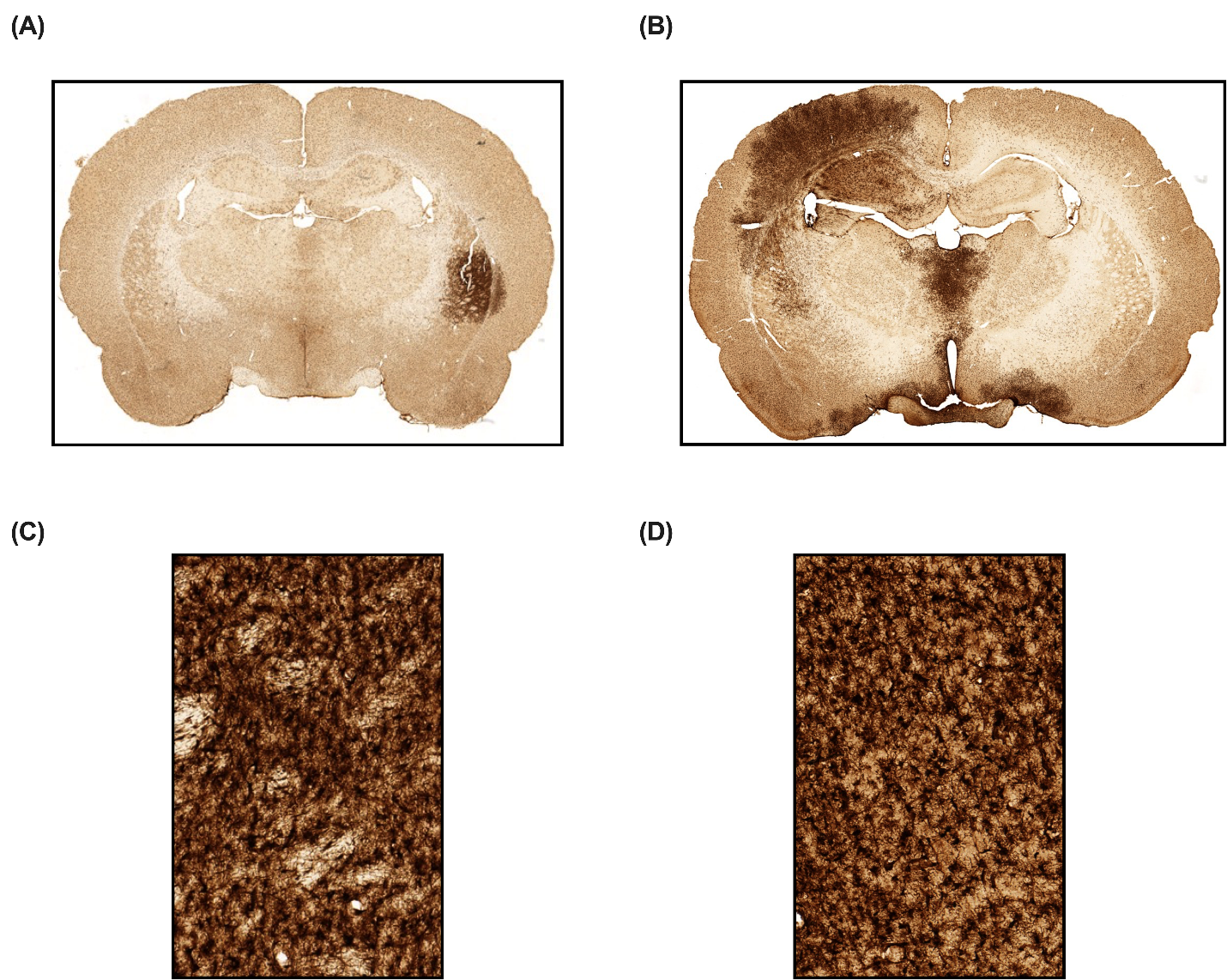
***

**Supplemental Figure 3.** “Microglia clusters.” (A) IBA1 stained section from Male Tg12099 +/- Sham animal. (B) IBA1 stained section from Male Tg12099 +/- rbTBI. (C) 10x image of cluster in A. (D) 10x image of cluster in cortex in B. rbTBI, repeated blast traumatic brain injury. Tg12099 +/-, transgenic heterozygous P301S Tau rat. IBA1, ionized calcium-binding adaptor molecule 1.
